# The disease burden of pertussis in adults 50 years old and older in the United States: a retrospective study

**DOI:** 10.1186/1471-2334-13-32

**Published:** 2013-01-23

**Authors:** Catherine Balderston McGuiness, Jerrold Hill, Eileen Fonseca, Gregory Hess, William Hitchcock, Girishanthy Krishnarajah

**Affiliations:** 1 IMS Health, One IMS Drive, Plymouth Meeting, Pennsylvania, PA, 19462, USA; 2 Leonard Davis Institute for Health Economics and Policy, University of Pennsylvania, 3641 Locust Walk, Pennsylvania, PA, 19104, USA; 3 La Jolla Pediatrics, 4520 Executive Drive, Suite 350, San Diego, CA, 92121, USA; 4 GlaxoSmithKline, One Franklin Plaza, Pennsylvania, PA, 19104, USA

**Keywords:** Pertussis, Whooping cough, Health economics, Adults, Elderly

## Abstract

**Background:**

While the incidence of pertussis has increased in adolescents and adults in recent years in the U.S., little is known about the incidence and economic burden of pertussis in older adults. This study provides evidence of the incidence of pertussis and direct medical charges associated with pertussis episodes of care (PEOCs) in adults aged 50 years and older in the U.S.

**Methods:**

PEOCs were divided into periods before and after the initial pertussis diagnosis was made (i.e., the index date) to capture any conditions immediately preceding the pertussis diagnosis that may have represented misdiagnoses and subsequent conditions that may have represented sequelae. Data were extracted from IMS's recently acquired SDI databases of longitudinal, patient-level practitioner claims and hospital operational billing records collected from private practitioners and hospitals, respectively, across the U.S. Patients 50 years and older with one or more ICD-9-CM diagnoses for pertussis/whooping cough and/or a laboratory test positive for Bordetella pertussis between 1/1/2006 and 10/31/2010 were eligible for study inclusion. Resource utilization and charges (i.e., unadjudicated claims) associated with the patient's physician and hospital care were analyzed. The nationally projected incidence of pertussis was estimated using a subsample of patients with the required data necessary for projection.

**Results:**

Estimated incidence of diagnosed pertussis ranged from 2.1-4.6 cases per 100,000 people across the two age groups (50–64 and [greater than or equal to] 65) during the years 2006 to 2010. The analysis of charges included 5,748 patients [greater than or equal to] 50 years of age with pertussis. Average charges across the entire episode of care were $1,835 and $14,428 per patient in the outpatient and inpatient settings, respectively. The average number of outpatient (i.e., private practitioner) visits was 2 per patient in both the pre-index and post-index periods.

**Conclusions:**

In the U.S., the incidence of diagnosed pertussis in adults 50 years and older has increased between 2006 and 2010. Healthcare utilization and charges associated with pertussis are substantial, suggesting the need for additional prevention and control strategies and a higher degree of clinical awareness on the part of health care providers. Additional research regarding pertussis in older populations is needed to substantiate these findings.

## Background

Pertussis (whooping cough) is a contagious respiratory illness caused by the bacterium *Bordetella pertussis*[1]. Before vaccines to prevent the disease were available, pertussis was a major cause of morbidity and mortality in U.S. children. The historical information regarding pertussis in adults suggests that cases were rare and/or underreported. Following the universal recommendation of diphtheria, whole-cell pertussis and tetanus (DPT) vaccines in U.S. children, a sharp decline in cases was observed for many decades. In recent decades, however, reported pertussis cases have been steadily increasing in the U.S. where pediatric vaccination rates remain high [2,3]. Additionally, the age range of affected individuals appears to have widened and reported cases have disproportionately increased in adolescents and adults [4-6]. In the U.S., pertussis now represents the least well controlled of all bacterial vaccine-preventable diseases for which universal childhood immunization is recommended [7,8].

Unlike many diseases for which immunization is recommended, neither a completed immunization series in childhood nor natural infection confer lifelong immunity [9-11], and thus poses additional challenges for prevention and control. Reported duration of immunity varies widely between studies but may be shorter than previously thought [9,12,13]. Pertussis susceptibility and infection in adults is a concern not only with respect to morbidity but also for the potential transmission risk to non-immune infants who are particularly vulnerable to complications and mortality [14]. Indeed, adults are an important source of B. pertussis infection in infants and children [15].

Until 2005, pertussis-containing vaccines were not indicated for use in adolescents or adults and only very recently (i.e., 2010) was a pertussis-containing vaccine recommended for adults over the age of 64. The Advisory Committee on Immunization Practices (ACIP) currently recommends that a one-time dose of Tdap (tetanus toxoid, reduced diphtheria toxoid, and acellular pertussis) vaccine is administered to all adults who have not received Tdap previously or for whom vaccine status is unknown to replace one of the 10-year tetanus and diphtheria (Td) boosters, and as soon as feasible to the following groups of adults who have not received Tdap previously or for whom vaccine status is unknown: 1) pregnant (>20 weeks gestation) or postpartum women, 2) close contacts of infants younger than age 12 months (e.g., grandparents and child-care providers), and 3) health-care personnel [16].

The recent resurgence of pertussis is well established [1,2,11,14,17,18] and while it appears that a wider age range is affected, little is known about the incidence or burden of pertussis in adults and the elderly. An understanding of the incidence and burden of pertussis in these populations is necessary for developing effective clinical approaches and public health programs to curb the spread of pertussis.

The primary objective of this study was to estimate the incidence and describe the burden of pertussis in a population that was not traditionally thought of as being affected by the disease (i.e., older adults). We examined the clinical presentation of and physician coding behaviors associated with pertussis as well as assessed reported charges from pertussis-related inpatient and outpatient healthcare utilization.

## Methods

A retrospective, observational study was conducted to estimate the incidence of diagnosed pertussis among adults 50 years and older in the U.S. and obtain descriptive data on the clinical burden and healthcare associated charges of pertussis episodes of care in this population.

### Data sources

Data were extracted from IMS’s U.S. databases, recently acquired from SDI, including private practitioner medical claims, hospital charge detail master data (a database of patient-level accounting records), and commercial outpatient laboratory test results. IMS data are accessible to researchers through grants, academic discounts and for general purchase.

The *private practitioner medical claims database* (CMS-1500) captures diagnoses and procedures across all types of payers for insured patients. This database comprises approximately 1 billion entries and over 80 million claims per year (~ two-thirds of all electronically filed medical claims in the U.S.) submitted by more than 870,000 physicians per month. Between 1/1/2006 and 10/31/2010, over 102 million unique patients aged 50 years old or older were observed in the private practitioner medical claims database. *Hospital charge detail master (CDM) data* are daily transactional patient charges from over 450 hospitals, representing over 9% of all nonfederal, acute and short-stay hospitals in the U.S. Between 1/1/2006 and 10/31/2010, over 35 million unique patients aged 50 years old or older were observed in the hospital database. Available data attributes include detailed drug, procedure, device, diagnosis, and applied charges information for each patient’s stay. In addition, patient demographics and admission/discharge characteristics are available. Data were also extracted from commercial outpatient laboratory test results representing approximately 40% of all commercial laboratory testing performed in the U.S. Patient-level data in this study were de-identified. All databases utilized in the current study were certified as being compliant with the Health Insurance Portability and Accountability Act (HIPAA).

### Samples

#### Unweighted sample

To assess healthcare utilization and charges, patients with at least one of the ICD-9-CM pertussis/whooping cough diagnoses [033.0 (whooping cough due to *B. pertussis*), 033.9 (whooping cough, organism unspecified), 484.3 (pneumonia in whooping cough)] or a positive laboratory test for *B. pertussis* (i.e., Polymerase Chain Reaction; PCR, antigen detection, culture or single sera IgA, IgG, and or IgM titers) between 1/1/2006 and 10/31/2010 were eligible for inclusion. If more than one inclusion criterion was present on the index date (i.e., the first confirmation ─ diagnosis or positive laboratory result ─ of pertussis within the study period), the following hierarchy was applied: hospital diagnosis, private practitioner diagnosis with preferred order 033.0, 033.9, then 484.3, and lastly positive laboratory result.

Patients with a positive *B. pertussis* laboratory test result were indexed to the date of specimen collection that yielded the pertussis-positive result. These patients must also have had a medical claim (from a hospital or private practitioner’s office) with a pertussis-related diagnosis and a date of service within 14 days of the collection of the laboratory test specimen to be included. Diagnoses were considered to be pertussis-like or related based on existing literature, clinical opinions, and/or internal data distributions of diagnoses made during the periods immediately before and immediately following the pertussis diagnosis. Examples of pertussis-like diagnoses included cough and upper and lower respiratory tract infections and pertussis related diagnoses included fractured ribs.

To ensure patients were observable throughout the duration of their episode of care, the study required observed healthcare activity from at least 3 months before to at least 3 months after the index date for each patient indexed to a private practitioner (CMS-1500) claim. Likewise, to ensure stability of the data reporting source (i.e., the practitioner or hospital), consistent claim submission during pre-index and post-index periods was required. Additional patient inclusion criteria included a known gender and age of 50 years or older at the time of initial diagnosis (i.e., the index date).

#### Weighted sample

Pertussis incidence was defined as the nationally projected number of patients with a new pertussis diagnosis (i.e., 033.0, 033.9 and/or 484.3) from the private practitioner database. The projected incidence was calculated using IMS’s medical data projection weights (see also Toback et al. [19]) to provide a nationally representative estimate of pertussis cases diagnosed in private practitioner offices between 2006 and 2010. The weights were estimated by month and physician specialty strata. The universe of office-based American Medical Association (AMA) practitioners (contained in the AMA master file [20]) was divided by the corresponding number of practitioners in the IMS sample. To ensure accuracy of the weighted incidence estimates, only practitioners whose sample activity was deemed representative of their specialty and who had relevant longitudinal continuity were included in the sample. Private practitioners whose data was used to create projection factors during the period of study represented between 9% (annual average in 2006) and 17% (annual average in 2010) of all general practice, internal medicine, and geriatric specialists in the nation. Incidence rates per 100,000 persons were calculated by dividing the nationally projected number of diagnosed pertussis cases by the U.S. Census populations [21] according to age group and year and multiplying the product by 100,000. Estimated incidence rates were compared to incidence rates of pertussis in the U.S. population aged 65 years old and older reported to the Nationally Notifiable Disease Surveillance System (NNDSS). The comparison was expressed as a ratio of the IMS incidence rate to the NNDSS rate by year from 2006 through 2010.

### Data analysis

To capture resource utilization and charges likely associated with pertussis (i.e., pertussis-like illness) before the initial diagnosis, we examined the frequency of each patient’s medical visits during the six month period before pertussis was diagnosed and used a hierarchical linear mixed model to compute a patient-specific cutoff which was one standard deviation above the patient’s mean interval (days) between visits. Visits for pertussis-like illness occurring between the index date and this individual cutoff defined the pre-index period for each patient. The end of each patient’s episode was defined as the visit date that preceded a 30-day period free of any pertussis-related diagnosis. Episode lengths were capped at 180 days based on the clinical opinion that pertussis episodes of care should resolve within 6 months and that longer episodes were likely be indicative of chronic/underlying conditions. Nearly all patients (99.5%) had episodes of care equal to or less than 180 days.

A descriptive analysis of demographic and health status information was undertaken. The unit of analysis was the patient and as these results were reported at the raw, observed level, they cannot be assumed to be nationally representative. Counts of patients and the percent of the total per category were summarized for demographic and health status variables. Analysis of Variance (ANOVA) was used to assess differences in patient characteristics of interest. All post hoc tests employed Bonferroni adjustments for multiple comparisons. Additional measures of central tendency and variability were calculated as appropriate. Resource utilization and charges were summarized in two patient age groups: 50–64 years and 65 years and older. The total sample was divided into the following three groups based on the setting in which the data/encounters were observed: private practitioner only, hospital only, and the combination. These descriptors inform as to the data being presented (e.g., if a patient is observable within the context of both private practitioner and hospital data, only the relevant data is displayed per setting). Not all patients were observable in both settings.

For assessment of outcomes associated with pertussis and physician coding behaviors for pertussis, all diagnosis and procedure codes were extracted from each unique patient’s record between the patient’s pertussis episode of care (PEOC) start and end dates. Descriptive analyses, including measures of central tendency and variability, were reported as appropriate (e.g., mean length of inpatient stay). The following outcomes and associated variables were reported in both the pre-index and post-index periods: outpatient visits (physician office and outpatient hospital), emergency department visits, hospital admissions, length of inpatient stay, intensive care unit admission, assisted ventilation, acute respiratory distress syndrome, and post-index death.

Resource utilization was also summarized by PEOC for the pre-index and post-index periods by age group (i.e., 50–64 years and 65 years and older). Predefined diagnoses and procedures associated with each encounter type (i.e., outpatient private practitioner offices plus hospital outpatient clinics and sites; hospitalizations plus emergency department visits) were reported. Mean visits per patient were also reported for each encounter type.

In assessing the reported charges associated with inpatient and outpatient healthcare utilization, total reported charges for each diagnostic group and point of care setting were calculated along with descriptive statistics. Charges for visits, diagnostic imaging, laboratory tests, outpatient respiratory/nebulizer procedures, and inpatient medications were reported. The mean charge per quantity within a specific category (e.g., visits, diagnostic imaging, asthma medications, etc.) was used to replace data points where charge values were greater than 3 SD from the mean or missing. All charges were adjusted to 2010 U.S. dollars, using the Medical Care Consumer Price Index for All Urban Consumers (CPI-U), specifically the medical service, inpatient hospital service, and outpatient hospital service indices.

## Results

### Unweighted Sample

The sample included 5,748 patients aged at least 50 years with pertussis, of whom 5,490 were observed in the private practitioner setting; 643 were observed in the hospital setting; and 385 were observed in both settings. The latter 385 patients were included in analyses for the combined settings (private practitioner plus hospital) as well as in the individual private practitioner and hospital strata.

Twenty four patients were excluded from the analysis due to unknown gender and 1,691 patients were excluded from the analysis because they were not observed in the database at least three months before the index date and either at least 3 months after the index date or with a record of death, reducing our confidence of complete data capture. A valid age was associated with each patient in the sample. Additionally, 2,651 patients with a positive pertussis laboratory test were excluded from the analysis because they lacked a corresponding medical claim for a pertussis related diagnosis during the 14 days before or after specimen collection.

Approximately two-thirds of patients within the total sample were female [*Χ*^2^(1,N = 5,748) = 698.68, p < .0001] (Table 1). Most patients were from the southern and western U.S. census regions. ANOVA results indicated patient age was significantly different according to the type of first confirmation of pertussis (i.e., ICD-9 code 033.0, 033.9, 484.3 or a positive pertussis laboratory test) [F(3, 5744) = 47.42, p < .0001]. Bonferroni adjusted post hoc comparisons revealed a significantly older group of patients whose initial pertussis confirmation was a diagnosis of pneumonia in whooping cough (i.e., ICD-9 code 484.3; see Table 2). Length of episode of care was also significantly different [F(3,5744) = 47.42 , p < .0001] with patients whose first confirmation of pertussis was a positive laboratory test having longer episodes of care (Table 2).

**Table 1:** Demographics and baseline clinical characteristics

	**Private practitioner setting**		**Hospital setting**		**Patients in both private practitioner and hospital settings**	
	**Patient count**	**% of Total**	**Patient count**	**% of Total**	**Patient count**	**% of Total**
**N**	**5,490**	100	**643**	**100**	**385**	**100**
**Gender**						
Male	1,779	32.4	238	37.0	145	37.7
Female	3,711	67.6	405	63.0	240	62.3
**Age, years**						
50-64	3,523	64.2	402	62.5	242	62.9
50-54	1,276	23.2	132	20.5	76	19.7
55-59	1,175	21.4	132	20.5	75	19.5
60-64	1,072	19.5	138	21.5	91	23.6
≥65	1,967	35.8	241	37.5	143	37.1
65-69	731	13.3	89	13.8	54	14.0
70-74	503	9.2	67	10.4	42	10.9
≥75	733	13.4	85	13.2	47	12.2
Mean (SD) Age	63 (9)	na	63 (9)	na	61 (9)	na
**US Census Region**						
Midwest	1,042	19.0	96	14.9	54	14.0
Northeast	664	12.1	165	25.7	71	18,4
South	2,236	40.7	218	33.9	130	33.8
West	1,547	28.2	164	25.5	130	33.8
**Payer Type**						
Commercial	4,137	75.4	467	72.6	281	73.0
Medicaid	87	1.6	26	4.0	16	4.2
Medicare	1,264	23.0	141	21.9	86	22.3
**Year of Initial Diagnosis**						
2006	570	10.4	63	9.8	21	5.5
2007	659	12.0	63	9.8	30	7.8
2008	861	15.7	86	13.4	55	14.3
2009	1,587	28.9	203	31.6	132	34.3
2010 (Jan-Oct)	1,813	33.0	228	36.5	147	38.2
**Length of Episode of Care, days**						
Mean (SD)	26 (32)	na	28 (34)	na	34 (37)	na
Median (range)	16 (1–180)	na	15 (1–180)	na	23 (1–180)	na

na = not applicable.

Census region information was not available for the entire study sample. A small percentage of patients had an unknown or other (e.g., TRICARE) insurer. Therefore, patient counts above do not always sum to the total population per setting.

**Table 2:** **Mean (95**% **confidence interval) age and episode of care length by type of initial pertussis confirmation**

**Initial pertussis confirmation**	**Age, years**	**Episode of care length, days**
ICD-9 code 033.0 (whooping cough due to B. pertussis ), N = 573	61.30 (60.56-62.03)	21.41 (19.13-23.70)
ICD-9 code 033.9 (whooping cough, organism unspecified), N = 2,231	63.07 (62.70-63.47)	21.35 (20.14-22.55)
ICD-9 code 484.3 (pneumonia in whooping cough), N = 233	69.43 (68.01-70.86)	21.82 (17.61-26.03)
Lab test positive for B. pertussis , N = 2,711	61.74 (61.42-62.06)	31.17 (29.93-32.42)

### Incidence of pertussis (weighted sample)

**Figure 1:**
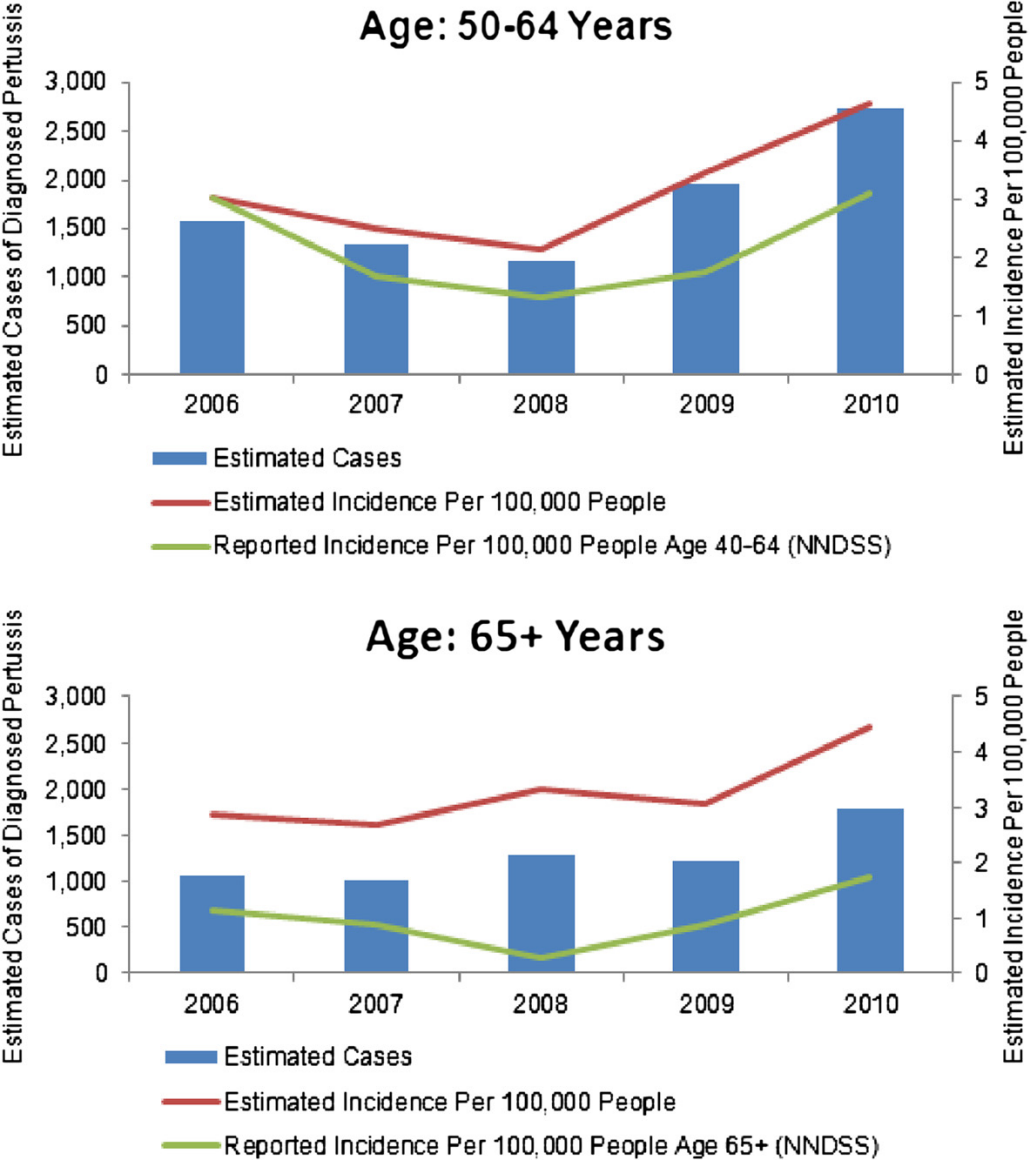
Estimated incidence of diagnosed pertussis (ICD-9 codes 033.0, 033.9, or 484.3) and reported incidence of pertussis from NNDSS.

Estimated national incidence of diagnosed pertussis ranged from 2.1-4.6 cases per 100,000 people across the two age groups (50–64 and ≥65) during the years 2006 to 2010 (Figure 1). Incidence per 100,000 increased over time from 3.0 in 2006 to 4.6 in 2010 for patients 50–64 years and from 2.9 in 2006 to 4.4 in 2010 for patients 65 years or older. The ratio of IMS to NNDSS rates from the ≥65 age group ranged from 2.86:1.15 (in 2006) to 3.31:0.28 (in 2008).

### Diagnoses associated with pertussis

During the pre-index period, cough and bronchitis (categorized as part of the larger lower respiratory tract infection group) were the initial diagnoses most frequently made before the pertussis diagnosis (Table 3). The majority of patients who presented with a pertussis-like condition (e.g., cough) in the pre-index period had an acute respiratory diagnosis (80% to 91%, depending on healthcare setting).

**Table 3:** Most common diagnosis pre- and post-index, private practitioner setting

**Diagnoses**	**Patient count**	**% of Total**
**Pre-Index Period**		
**Total Unique Patients in the Population**	**5,490**	
**Patients with ≥ 1 Pertussis-like event**	**3,140**	**57**
**Acute Respiratory:**	**2,857**	**52**
COUGH	1,350	25
UPPER RESPIRATORY TRACT INFECTION	1,004	18
ACUTE BRONCHITIS	938	17
ASTHMA	449	8
ALLERGY	413	8
LOWER RESPIRATORY TRACT INFECTION	387	7
OTHER PNEUMONIA	313	6
**Other :**	**1,798**	**33**
HYPERTENSION	579	11
SHORTNESS OF BREATH	368	7
HIGH CHOLESTEROL	316	6
GERD	290	5
CHEST PAIN	266	5
COPD AND OTHER ALLIED CONDITIONS	229	4
DIABETES	224	4
LUMBAR STRAIN OR BACK PAIN	171	3
**Post-Index Period**		
**Total Unique Patients in the Population**	**5,490**	**100**
**Acute Respiratory:**	**5,053**	**92**
WHOOPING COUGH-UNSPECIFIED ORGANISM	2,224	41
COUGH	2,222	40
UPPER RESPIRATORY TRACT INFECTION	770	14
ACUTE BRONCHITIS	694	13
WHOOPING COUGH-BORDETELLA PERTUSSIS	605	11
ASTHMA	551	10
OTHER PNEUMONIA	468	9
ALLERGY	404	7
LOWER RESPIRATORY TRACT INFECTION	359	7
PNEUMONIA IN WHOOPING COUGH	237	4
**Other:**	**2,309**	**42**
HYPERTENSION	851	16
HIGH CHOLESTEROL	495	9
SHORTNESS OF BREATH	445	8
DIABETES	421	8
GERD	421	8
COPD AND OTHER ALLIED CONDITIONS	388	7
CHEST PAIN	300	5

Patients may be counted in multiple types of diagnoses. Therefore, the sum of the sub-classifications may total more than the total unique patients.

Diagnoses in >2% of patients are listed.

During the post-index period, pertussis and cough were frequent diagnoses (Table 3). Additionally, in the private practitioner setting, pneumonia diagnoses were observed in 10% and 19% of patients 50–64 and ≥65, respectively. Within the hospital setting, pneumonia diagnoses were observed in 10% of patients in the younger age group and 17% of patients in the older age group. Of patients that were observed in both private practitioner and hospital settings, approximately 15% and 31% of patients aged 50–64 and ≥ 65, respectively, were diagnosed with pneumonia.

### Healthcare charges and utilization

The average medical charges associated with diagnosed pertussis ranged from $496 to $3,239 per patient in the pre-index period and $987 to $16,971 per patient in the post-index period. Table 4 shows data from all patients grouped by the healthcare setting. Per patient resource use and charges were consistently higher during the post-index period than the pre-index period (Table 4). During the pre-index period, average per patient charges were slightly higher in the subgroup of 50 to 64 year old patients than in the sample as a whole or in the subgroup ≥ 65 year old patients; in the post-index period, the average per patient charges for 50–64 year old patients tended to be higher than those for ≥ 65 year old patients, particularly among the combined private practitioner and hospital patient group (Table 4). Additional analysis found one or more patients with disproportionately high utilization of healthcare resources in the subgroup of 50 to 64 year old patients which led to the observed increases in charges. During the post-index period, among patients 50 to 64 years old, average per patient charges were $1,533 for those in the private practitioner setting, $16,971 for those in the hospital setting, and $15,062 for those represented in both settings. During the post-index period, among patients ≥ 65 years, for the same respective categories, the average per patient charges were $987, $10,693, and $7,102. During both the pre- and post-index periods, visit charges and inpatient/ER medications dispensed constituted the bulk of the detailed charges (Table 4).

**Table 4:** Healthcare resource utilization and charges observed during the pre-index and post-index periods by healthcare setting

	**Both private practitioner and hospital patient mean (SE)**			**Private practitioner only patient mean (SE)**			**Hospital only patient mean (SE)**		
	**Total**	**50-64 Years**	**>65 Years**	**Total**	**50-64 Years**	**>65 Years**	**Total**	**50-64 Years**	**>65 Years**
	**(N = 385)**	**(N = 242)**	**(N = 143)**	**(N = 5,490)**	**(N = 3,523)**	**(N = 1,967)**	**(N = 643)**	**(N = 402)**	**(N = 241)**
**Panel A: Pre-Index Resource Utilization**									
**Outpatient**									
Visits	2.1 (0.2)	2.4 (0.3)	1.6 (0.2)	1.6 (0.0)	2.7 (0.1)	1.7 (0.1)	0.2 (0.0)	0.2 (0.0)	0.3 (0.1)
Diagnostic imaging	0.3 (0.0)	0.4 (0.1)	0.3 (0.1)	0.3 (0.0)	0.3 (0.0)	0.3 (0.0)	0.1 (0.0)	0.1 (0.0)	0.1 (0.0)
Lab tests	0.3 (0.1)	0.3 (0.1)	0.2 (0.1)	0.3 (0.0)	0.3 (0.0)	0.3 (0.0)	0.0 (0.0)	0.0 (0.0)	0.0 (0.0)
Respiratory treatments	0.4 (0.1)	0.4 (0.1)	0.2 (0.1)	0.3 (0.0)	0.4 (0.0)	0.2 (0.0)	0.0 (0.0)	0.0 (0.0)	0.0 (0.0)
**Inpatient (including emergency department data)**									
Visits	0.1 (0.0)	0.1 (0.0)	0.1 (0.0)	na	na	na	0.1 (0.0)	0.1 (0.0)	0.1 (0.0)
Diagnostic imaging	0.1 (0.0)	0.1 (0.0)	0.1 (0.1)	na	na	na	0.1 (0.0)	0.1 (0.0)	0.1 (0.0)
Lab tests	0.1 (0.0)	0.1 (0.1)	0.0 (0.0)	na	na	na	0.1 (0.0)	0.0 (0.0)	0.1 (0.0)
Medications	9.8 (5.3)	10.5 (7.7)	8.7 (5.7)	na	na	na	9.5 (3.3)	7.4 (4.7)	12.9 (4.4)
Systemic antibiotics	1.2 (0.8)	1.2 (1.1)	1.3 (1.2)	na	na	na	0.9 (0.5)	0.7 (0.7)	1.1 (0.8)
Asthma	0.8 (0.4)	0.8 (0.5)	0.6 (0.6)	na	na	na	0.7 (0.3)	0.6 (0.3)	0.8 (0.4)
All other medications	7.9 (4.3)	8.5 (6.1)	6.8 (4.9)	na	na	na	8.0 (2.8)	6.1 (3.7)	11.0 (3.9)
**Panel B: Pre-Index Charges, 2010 Dollars**									
**Outpatient**									
Visits	420 (37.4)	470 (49.0)	331 (55.6)	225 (9.4)	214 (6.5)	244 (23.5)	228 (25.9)	226 (29.7)	230 (48.4)
Diagnostic imaging	50 (7.2)	55 (9.0)	43 (12.0)	35 (1.9)	38 (2.5)	30 (2.9)	38 (6.3)	37 (6.0)	39 (13.7)
Lab tests	15 (6.0)	19 (9.3)	9 (3.8)	12 (0.9)	13 (1.1)	10 (1.4)	4 (1.7)	5 (2.6)	2 (1.5)
Respiratory treatments	25 (4.7)	31 (6.8)	14 (5.2)	19 (1.2)	23 (1.7)	13 (1.5)	0 (0.0)	0 (0.0)	0 (0.0)
All other	371 (101.4)	434 (140.3)	263 (134.6)	76 (4.7)	76 (5.7)	78 (8.4)	345 (83.4)	312 (92.3)	399 (160.9)
All Outpatient Charges	932 (130.1)	1,080 (172.7)	683 (192.2)	497 (48.4)	496 (62.0)	499 (77.2)	892 (103.3)	907 (117.9)	868 (193.6)
**Inpatient (including emergency department data)**									
Visits	284 (115.9)	296 (135.8)	265 (211.8)	na	na	na	322 (81.6)	270 (87.0)	409 (162.5)
Diagnostic imaging	38 (15.8)	33 (15.6)	48 (33.5)	na	na	na	38 (10.1)	33 (10.2)	48 (20.9)
Lab tests	12 (9.6)	18 (15.2)	2 (1.8)	na	na	na	9 (5.8)	11 (9.1)	5 (2.7)
Medications	364 (214.3)	337 (271.9)	410 (349.5)	na	na	na	0 (0.0)	229 (164.2)	409 (220.0)
Systemic antibiotics	160 (106.7)	118 (107.3)	231 (223.0)	na	na	na	296 (131.6)	71 (64.6)	177 (135.1)
Asthma	27 (13.9)	30 (17.9)	22 (22.2)	na	na	na	111 (64.7)	19 (10.8)	25 (14.5)
All other medications	177 (102.7)	189 (150.5)	157 (108.2)	na	na	na	21 (8.7)	138 (91.5)	207 (80.0)
All other charges	691 (293.0)	722 (406.6)	639 (387.9)	na	na	na	164 (64.5)	557 (248.5)	1,500 (502.3)
All Inpatient Charges	1,390 (635.5)	1,406 (836.0)	1,364 (966.1)	na	na	na	1,576 (446.2)	1,099 (509.9)	2,372 (832.0)
All Inpatient and Outpatient Charges	2,323 (661.5)	2,485 (878.3)	2,048 (984.6)	na	na	na	2,468 (469.9)	2,006 (540.6)	3,239 (870.1)
**Panel C: Post-Index Resource Utilization**									
**Outpatient**									
Visits	2.9 (0.2)	3.0 (0.3)	2.8 (0.3)	2.4 (0.0)	2.3 (0.1)	2.5 (0.1)	0.5 (0.1)	0.5 (0.1)	0.6 (0.1)
Diagnostic imaging	0.5 (0.1)	0.5 (0.1)	0.4 (0.1)	0.4 (0.0)	0.4 (0.0)	0.4 (0.0)	0.2 (0.0)	0.2 (0.0)	0.2 (0.0)
Lab tests	0.6 (0.1)	0.6 (0.1)	0.5 (0.1)	0.6 (0.0)	0.7 (0.0)	0.5 (0.0)	0.3 (0.0)	0.3 (0.1)	0.3 (0.1)
Respiratory treatments	0.5 (0.1)	0.6 (0.1)	0.4 (0.1)	0.5 (0.0)	0.5 (0.0)	0.4 (0.0)	0.0 (0.0)	0.0 (0.0)	0.0 (0.0)
**Inpatient (including emergency department data)**									
Visits	0.3 (0.0)	0.3 (0.1)	0.3 (0.0)	na	na	na	0.4 (0.0)	0.4 (0.0)	97 (0.0)
Diagnostic imaging	0.6 (0.2)	0.7 (0.3)	0.4 (0.1)	na	na	na	0.7 (0.1)	0.8 (0.2)	134 (0.1)
Lab tests	0.9 (0.2)	0.9 (0.3)	0.9 (0.3)	na	na	na	1.0 (0.1)	0.9 (0.2)	261 (0.2)
Medications	59.9 (15.3)	72.5 (23.5)	38.5 (10.1)	na	na	na	95.9 (24.7)	114.8 (38.9)	15,497 (10.9)
Systemic antibiotics	6.4 (2.9)	8.8 (4.6)	2.4 (0.7)	na	na	na	6.1 (1.8)	7.3 (2.9)	981 (0.8)
Asthma	7.5 (2.4)	8.5 (3.7)	5.7 (1.8)	na	na	na	6.6 (1.5)	6.5 (2.3)	1,593 (1.3)
All other medications	46.0 (11.2)	55.2 (17.1)	30.3 (7.2)	na	na	na	83.2 (23.4)	100.9 (37.0)	12,923 (9.6)
**Panel D: Post-Index Charges, 2010 Dollars**									
**Outpatient**									
Visits	626 (45.9)	632 (59.0)	615 (72.8)	331 (7.5)	324 (8.8)	344 (13.5)	489 (45.5)	469 (44.5)	523 (95.7)
Diagnostic imaging	91 (13.8)	82 (15.3)	107 (26.8)	52 (2.5)	56 (3.3)	46 (3.6)	51 (6.6)	44 (6.4)	61 (13.7)
Lab tests	36 (5.3)	38 (6.6)	33 (8.9)	26 (1.4)	31 (2.1)	17 (1.4)	29 (4.9)	32 (6.6)	25 (7.2)
Respiratory treatments	39 (7.3)	48 (10.7)	25 (7.8)	32 (1.8)	37 (2.5)	23 (2.2)	0 (0.0)	0 (0.0)	0 (0.0)
All other	514 (112.5)	460 (113.5)	603 (233.2)	94 (50.0)	101 (6.8)	82 (6.8)	600 (101.7)	560 (102.1)	668 (210.2)
All Outpatient Charges	1,436 (167.0)	1,378 (181.1)	1,519 (329.7)	1,337 (195.9)	1,533 (299.3)	987 (106.7)	1,576 (146.0)	1,554 (146.2)	1,613 (304.2)
**Inpatient (including emergency department data)**									
Visits	2,162 (413.2)	2,533 (624.7)	1,535 (343.3)	na	na	na	2,460 (327.6)	2,427 (472.6)	2,516 (376.4)
Diagnostic imaging	157 (31.0)	184 (46.6)	111 (26.9)	na	na	na	230 (44.9)	256 (68.8)	186 (33.9)
Lab tests	127 (32.4)	149 (49.1)	90 (26.6)	na	na	na	140 (23.0)	145 (34.0)	133 (23.3)
Medications	2,106 (595.6)	2,917 (937.4)	734 (195.1)	na	na	na	2,652 (615.5)	3,423 (972.8)	1,366 (233.6)
Systemic antibiotics	422 (139.2)	571 (218.7)	171 (54.6)	na	na	na	530 (140.6)	646 (220.5)	338 (72.2)
Asthma	182 (52.3)	209 (78.2)	136 (48.3)	na	na	na	155 (32.7)	160 (48.2)	147 (33.7)
All other medications	1,502 (443.9)	2,137 (699.6)	427 (125.5)	na	na	na	1,967 (479.0)	2,617 (757.9)	881 (186.0)
All other charges	6,117 (1,752.1)	7,893 (2,745.5)	3,112 (773.4)	na	na	na	7,560 (1,742.8)	9,167 (2,748.1)	4,881 (759.1)
All Inpatient Charges	10,669 (2,710.9)	13,675 (4,237.2)	5,582 (1,284.0)	na	na	na	13,042 (2,627.6)	15,417 (4,122.6)	9,080 (1,342.5)
All Inpatient and Outpatient Charges	12,105 (2,732.5)	15,062 (4,265.6)	7,102 (1,349.0)	na	na	na	14,618 (2,633.4)	16,971 (4,128.3)	10,693 (1,376.0)

## Discussion

To the authors’ knowledge, this study is the first to describe a large sample of adults 50 years and older with pertussis. Consistent with prior findings [22-28], our results suggest that under-reporting of pertussis cases occurs despite nationwide mandates to notify health authorities when cases are identified. We estimated the national incidence of diagnosed pertussis to be between 2.1 and 4.6 cases per 100,000 people across the two age groups (50–64 and ≥65) during the years 2006 to 2010. By contrast, the ratios of IMS to NNDSS incidence rates were greater than one in each of the five years analyzed supporting the hypothesis that pertussis infections in older adults occur more frequently than previously reported. Although larger than NNDSS estimates, we suspect our estimated incidence of diagnosed pertussis is still an understatement of the true rate of diagnosed pertussis cases as patients who tested positive for pertussis but did not have a private practitioner claim with an ICD-9-CM code for pertussis and patients that were only observed in the hospital setting were not accounted for in the incidence calculation. While infants and young children bear a well-documented burden caused by Bordetella pertussis, this disease’s direct financial and health burden is not exclusive to childhood as once previously thought. The increasing incidence of pertussis among the older adults in this study is consistent with studies in adolescents and younger adults, among whom the incidence of pertussis also appears to be increasing [11,14-18].

Our observation that females with pertussis represented a significantly higher proportion of the total sample compared to males is consistent with results in younger patient populations [22,23] and behavioral (i.e., treatment-seeking) literature [29,30]. The age effect on the initial diagnosis of pneumonia in whooping cough suggests that susceptibility to pneumonia and/or a pneumonia diagnosis may increase as age increases. Regarding the finding of significantly longer episodes of care among patients whose initial pertussis confirmations were made through positive laboratory tests, we interpret that this is related to severity. That is, a sicker patient may be more likely to undergo laboratory testing.

The results of this study also suggest that pertussis is not always diagnosed on the first occasion that the patient presents for care. This finding has been previously reported [e.g., [27,28]. Cough and acute bronchitis were the most common conditions diagnosed prior to the pertussis diagnosis. Over half (57%) of the sample had one or more visits with at least one pertussis-like diagnosis in the period immediately preceding the index pertussis diagnosis. These initial diagnoses may have been made while practitioners awaited laboratory confirmation of etiology or they may have been misdiagnoses. If the latter is true, misdiagnosed patients that remain untreated represent a potential source of transmission to others. Under diagnosis of pertussis in older patients may occur because healthcare providers continue to view pertussis as a childhood disease [14]. These results underscore the need to heighten healthcare provider awareness of the occurrence of pertussis in adults, including older adults.

The average charges associated with a diagnosed pertussis episode of care ranged from $496 to $3,239 per patient in the pre-index period and $987 to $16,971 per patient in the post-index period. When the average episode of care charges from patients observed in the private practitioner setting ($1,834) are applied to the average annual incidence estimate between 2006 and 2010 (1,513 cases), the extrapolated value approaches 3 million U.S. dollars. Within some categories (e.g., visits, lab tests, medications) the observed charges were greater in the younger age group (i.e., 50–64 years old) compared to the older (i.e., 65+ years old) age group. This result was driven by a minority of patients in the 50–64 year old group with very high healthcare utilization (and thus charges). Additionally, there were more patients in the younger group with asthma which may have led to the observation of increased utilization of respiratory/nebulizer treatments and medications. It is also possible that physicians are more likely to order lab tests for younger patients or that younger patients delay seeking care for a longer period of time and as a result present with more severe cases necessitating additional healthcare resources.

The use of charges is a proxy for the direct medical costs and represents the upper bound of the direct cost of physician office and hospital encounters from the current study. Historical direct medical costs (i.e., 2002 US$) of adult pertussis cases in the U.S. obtained from published literature range from $181 to $5,310 per patient depending on a number of factors including geographic location, population and type of services examined [31,32]. Additionally, while not studied here, the indirect costs for the patients and their caregivers should also be considered in the societal economic burden.

The results of this study are subject to at least four limitations. First, with the exception of the projected incidence rates of ICD-9-CM coded pertussis from private practitioners, the data analyzed in the current study may not be generalizeable to the entire nation as it is an unweighted convenience sample obtained for the purpose of describing pertussis episodes of care. Second, charges were used as a measure of a portion of the economic burden. Because charges for healthcare services are typically higher than reimbursed amounts, charges reflected in the current study are likely to be higher than the costs paid by health insurers and patients. Third, claims data can be inherently limiting because they are collected for billing and reimbursement purposes, rather than for research purposes. This particular limitation may have yielded an underestimate of the projected incidence of diagnosed pertussis if practitioners are reluctant to submit a claim with a pertussis code in absence of laboratory confirmation, or ‘under’ code for other reasons. In addition, if a diagnosis were confirmed by a laboratory result after a claim was submitted, a physician would not submit a second claim as reimbursement would not be affected. Finally, it is likely that the methods used to diagnose pertussis (i.e., clinical and/or laboratory evaluations) in this study are not always 100% sensitive and/or 100% specific potentially yielding imprecise results. The retrospective, observational nature of the study also should be considered when interpreting the results as it could make the results subject to selection bias. To our knowledge, potential biases may include an underrepresentation of patients receiving care when a reimbursement claim is not submitted (e.g., patients without health insurance), underrepresentation of patients consulting providers that do not submit medical claims electronically as well as any miscoding, none of which could be identified or corrected for in this analysis. Strengths of the study include the large sample size and the national representativeness of the weighted sample and the inclusion of patient data from multiple care settings (i.e., private practitioners, hospitals, and laboratories).

## Conclusions

The results of this study suggest that the incidence of diagnosed pertussis is greater than what is reported through the national surveillance system (i.e., NNDSS), and appears to have increased over recent years among adults 50 years and older. The portion of the economic impact measured (i.e., charges) was between $1,835 and $14,428 over the episode of patient care from the time of first presentation with symptoms until clinical care was no longer sought. The results support the need for greater clinical awareness, as well as better prevention and control efforts to reduce the burden of pertussis in adults. Such efforts may, in turn, decrease the transmission to other populations where the potential for substantial morbidity and mortality exists (e.g., infants). Additional research in older adult populations is warranted to further substantiate the current findings.

## Competing interests

KBM, JH, EF, and GH were employees of SDI during the period in which this study was conducted. SDI has since been acquired by IMS Health and the aforementioned authors are now employees of IMS. SDI received funding from GlaxoSmithKline to conduct these analyses. GK is an employee of and owner of stock in GlaxoSmithKline, a manufacturer of a pertussis-containing vaccine. WH receives fees from GlaxoSmithKline for his work as a consultant, speaker and investigator.

## Authors’ contributions

KBM conceived of the study and its general design, performed the statistical analyses with respect to hypothesis testing, interpreted the results, and helped to draft the manuscript. JH participated in critical decisions regarding the study’s design, assisted with statistical analyses and interpretation of results, and edited the manuscript. EF conceived the technical specifications of the study’s design and assisted with their implementation, spearheaded the coordination of the study, and edited the manuscript. GH, GK, and WH participated in critical decisions regarding the study’s design, assisted with interpretation of results, and edited the manuscript. All authors have read and approved of the final manuscript.

## Pre-publication history

The pre-publication history for this paper can be accessed here:

http://www.biomedcentral.com/1471-2334/13/32/prepub
